# Mixed Peptide-Conjugated Chitosan Matrices as Multi-Receptor Targeted Cell-Adhesive Scaffolds

**DOI:** 10.3390/ijms19092713

**Published:** 2018-09-11

**Authors:** Kentaro Hozumi, Motoyoshi Nomizu

**Affiliations:** 1 Department of Clinical Biochemistry, School of Pharmacy, Tokyo University of Pharmacy and Life Sciences, Hachioji, Tokyo 192-0392, Japan; 2 Department of Applied Clinical Dietetics, Kitasato Junior College of Health and Hygienic Sciences, Minamiuonuma, Niigata 949-7241, Japan

**Keywords:** cell attachment, chitosan, extracellular matrix, integrin, peptide, scaffold

## Abstract

Biomaterials are important for cell and tissue engineering. Chitosan is widely used as a scaffold because it is easily modified using its amino groups, can easily form a matrix, is stable under physiological conditions, and is inactive for cell adhesion. Chitosan is an excellent platform for peptide ligands, especially cell adhesive peptides derived from extracellular matrix (ECM) proteins. ECM proteins, such as collagen, fibronectin, and laminin, are multifunctional and have diverse cell attachment sites. Various cell adhesive peptides have been identified from the ECM proteins, and these are useful to design functional biomaterials. The cell attachment activity of peptides is influenced by the solubility, conformation, and coating efficiency to solid materials, whereas immobilization of peptides to a polysaccharide such as chitosan avoids these problems. Peptide–chitosan matrices promote various biological activities depending on the peptide. When the peptides are immobilized to chitosan, the activity of the peptides is significantly enhanced. Further, mixed peptide–chitosan matrices, conjugated with more than one peptide on a chitosan matrix, interact with multiple cellular receptors and promote specific biological responses via receptor cross-talk. Receptor cross-talk is important for mimicking the biological activity of ECM and the proteins. The mixed peptide–chitosan matrix approach is useful to develop biomaterials as a synthetic ECM for cell and tissue engineering.

## 1. Introduction

Biologically active scaffolds are useful for cell and tissue engineering. Thus far, many natural polysaccharides, including alginate, cellulose, chitosan, hyaluronan, and starch, have been used as scaffold substrates [1,2,3]. Chitosan, a partially deacetylated chitin, is composed of (1-4)-2-acetoamido-2-deoxy-β-d-glucan (*N*-acetyl-d-glucosamine) and (1-4)-2-amino-2-deoxy-β-d-glucan (d-glucosamine) units [4,5,6]. Chitosan, a cationic polysaccharide, is soluble in an acidic solution but it is rarely soluble in a physiological pH solution. Chitosan has been incorporated into various matrices, such as fibers [7], films [8], sponges [9], particles [10,11,12], and hydrogels [13,14] by basically treatment with alkali or cross-linking. A chitosan matrix is stable under physiological conditions in vitro and gradually undergoes degradation by lysozyme or chitinase in vivo [15]. A chitosan matrix directly adheres to tissues but alone does not have cell attachment activity suggesting that this property has advantages as a scaffold substrate for the construction of cell-adhesive scaffolds [16,17].

The extracellular matrix (ECM) provides environments for cells and has various biological functions. ECM proteins, such as collagen (COL) [18,19], fibronectin (FN) [20], and laminin (LM) [21], are multifunctional and contain diverse biologically active sites. Many cell adhesive peptides, such as RGD (Arg-Gly-Asp) from FN and both IKVAV (Ile-Lys-Val-Ala-Val) and YIGSR (Tyr-Ile-Gly-Ser-Arg) from LM, have been identified within ECM proteins and promote cell attachment, migration, spreading, differentiation, and proliferation through specific cellular receptors, including integrins and syndecans [22,23,24]. These ECM-derived peptides are useful to analyze mechanisms involved in the cell attachment process [24,25]. Conjugation of peptides, such as the RGD sequence, to chitosan is a useful strategy to produce chitosan derivatives and to add biological function on the chitosan [26,27]. The RGD peptide, originally derived from FN, is well characterized and widely used to promote adhesion between substrates and cells [28,29]. Briefly, the RGD peptide has been conjugated on the chitosan to add cell attachment activity for cell culture [30], to add cell differentiation activity for tissue and organ culture [31], and to add cell surface recognition activity for a gene or drug delivery system [32]. For example, chitosan and its derivatives are one of the candidates for biosynthetic bone grafts with efficient mineralization for regeneration of fractured bones. Vaidya et al. reported that a chitosan/polyethylene oxide/polycaprolactone fiber mesh was suitable to bone regenerative applications, and a RGD-conjugated chitosan/polyethylene oxide/polycaprolactone fiber mesh promoted bone stromal cell attachment and spreading with metabolic activity and accelerated bone regeneration [33].

Peptide–chitosan systems could also be used in other fields of science. Costa et al. reported the use of the Dhvar5 antimicrobial peptide immobilized chitosan films [34]. They chose chitosan as an antimicrobial scaffold due to its antimicrobial properties and readiness to be functionalized. The Dhvar5–chitosan film effectively improved antimicrobial effect cause by controlled immobilization of peptide to the chitosan film. Furthermore, Mukherjee et al. reported that tooth slices treated with the leucine-rich amelogenin peptide within a chitosan hydrogel showed a dense mineralized layer consisting of highly organized enamel-like apatite crystals that was comparable with an amelogenin recombinant protein-containing chitosan mixture for generating a mineralized layer [35]. These results suggest that optimized peptides enhanced the biological activities of chitosan such as bone regeneration, antimicrobial activity, and tooth mineralization. Almost all peptide–chitosan systems have focused on a single biologically active peptide conjugated to chitosan.

Chitosan alone does not have cell attachment activity. Since the cell attachment activity of peptides is influenced by their solubility, conformation, and coating efficiency to solid materials, immobilization of peptides to a polysaccharide, such as chitosan, has advantages to avoid the problems. We previously identified more than a hundred cell-adhesive peptides from thousands of LM-derived peptides and demonstrated that peptide–chitosan matrix systems are a useful strategy to evaluate the cell attachment activity of peptides [36,37,38]. We chose chitosan for our immobilized peptide scaffolds because it is non-cell adhesive, stable, and biocompatible, and peptides can be immobilized on chitosan with the desired amount, correct sequence direction, and mixed ratios of different peptides can be immobilized. Of specific importance is that LM-derived cell attachment peptide–chitosan matrices show biological activity depending on the immobilized peptide(s) [36,37,38]. Further, when the peptides are immobilized to chitosan, the activity of the peptides is significantly enhanced compared with that of the peptides coated on plastic tissue culture plates [36].

The combination of cell-adhesive peptides bound to different cellular receptors is useful to analyze the cell attachment mechanisms and to mimic the biological functions of ECM proteins. For example, LMs are heterotrimeric basement membrane proteins that have multiple biological functions through interactions with other ECM molecules and with cell surface receptors [21,39,40]. LMs consist of α, β, and γ chains that assemble into a triple-stranded coiled-coil structure. At least 19 isoforms of LMs, consisting of five α, three β, and three γ chains, have been identified [40,41]. LMs have diverse biological activities, including promotion of cell adhesion, cell migration, neurite outgrowth, and tumor metastasis. Five different LM α chains are tissue- and/or developmental stage-specifically expressed. For example, the α1 chain is expressed in the blastocyst neuroectodermally-derived tissues and in developing kidney in the early embryo [42,43]. The α2 chain is expressed in both skeletal and cardiac muscle, peripheral nerve, brain, and capillaries [44]. The α3 chain is mainly localized in skin and in other epithelia [45,46]. The α4 chain is detected in the microvasculature and in smooth muscle [47,48], and the α5 chain is expressed in multiple tissues during development, in adult microvasculature, and in various epithelia [49,50]. The diversity of the α chain contributes critically to laminin isoform-specific functions [40]. LM-111 consists of α1, β1, and γ1 chains, and bind at least nine different cell surface receptors [51]. The data suggest that LM-111-mediated cell attachment may involve multiple sites on the molecule and multiple cell surface receptors. Mixtures of the different peptides can be immobilized on a chitosan matrix. The mixing ratio of different peptides is easily controlled, and a synergistic effect of the peptides is clearly observed. The biological activities of mixed peptide–chitosan matrices are altered by specific combinations and mixing ratios of peptides that act via the receptor cross-talk.

Many cell adhesive materials are commercially available, and almost all of these materials are based on extracted ECM proteins and recombinant proteins. These materials are useful to promote and analyze the cell attachment and differentiation mechanisms in vitro that are mediated by soluble molecules and/or whole proteins. ECM proteins are large and multifunctional molecules. Cell-adhesive scaffolds that conjugate the cell adhesive peptides, such as RGD, IKVAV, and YIGSR, to the plastic plate, nanofiber, and hydrogel are also available, but these scaffolds involve a single conjugated peptide. The mixed peptide–chitosan matrices are useful to analyze multi-receptor interactions and to develop biomaterials as a synthetic ECM. Here, we have focused on the mixed peptide–chitosan matrices and describe their application for analysis of cellular events provoked by cell attachment and for the development of biomaterials.

## 2. Preparation of Peptide–Chitosan Matrices

Chitosan and its derivatives are beneficial in cell culture, wound healing, and tissue engineering, and as antimicrobial agents and for gene or drug delivery systems. There are several methods to immobilize the peptide to the chitosan [52,53,54,55]. Ho et al. used 1-ethyl-3-(3-dimethylaminopropyl)-carbodiimide (EDC) and *N*-hydroxy succinimide (NHS) to immobilize the carboxyl residue of peptide to the amine residue of chitosan [53], Tigli et al. immobilized the amine residue of the peptide to the amine residue of chitosan using NHS and suberic acid bis (*N*-hydroxy-succinimide ester) [54], and Masuko et al. introduced 2-iminothiolane to the amine residue of chitosan and immobilized the cysteine-containing peptide by disulfide bond formation to the 2-iminothiolane–chitosan [55]. We introduced *N*-(*m*-maleimidobenzoyloxy) succinimide (MBS) to the amine residue of chitosan and immobilized the cysteine-containing peptides to the maleimidobenzoyl (MB) group through the mercapto group [56].

**Figure 1:**
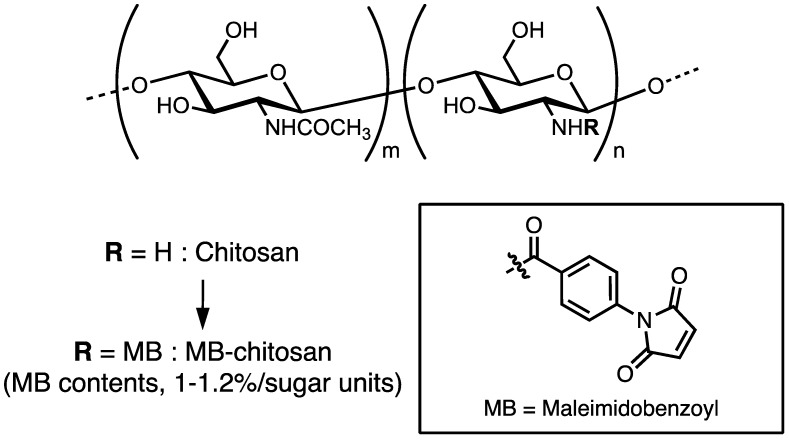
Preparation of maleimidobenzoyl (MB)–chitosan. Chitosan was reacted with MBS (*N*-(*m*-maleimidobenzoyloxy) succinimide) in dimethylformamide (DMF) solution. Substitution ratio of the MB groups to the chitosan was approximately 1–1.2%/sugar unit.

Briefly, chitosan (Chitosan 10, deacetylation rate: minimum 80.0 mol/mol%, Wako Pure Chemical or Chitosan low molecular weight 20–300 cP; deacetylation rate: 75–85 mol/mol%, Sigma-Aldrich, Saint Louis, MO, USA) was dissolved in 2% acetic acid (AcOH) and 25% dimethylformamide (DMF) solution and reacted with MBS by adjusting the pH using 4% ammonia solution. After washing the unreacted MBS, MB–chitosan was obtained, and the substitution ratio of the MB groups to the chitosan is 1.0–1.2%/sugar unit (Figure 1). MB–chitosan in 4% AcOH solution was added to the cell culture plates or slide glass chambers and dried for 2 days to coat the plates. Then, the plates or glass chambers were treated with 1 M NaHCO_3_ solution to form a matrix. For conjugation of the peptides to MB–chitosan, CGG (Cys-Gly-Gly)-peptides were synthesized by the 9-fluorenylmethoxycarbonyl (Fmoc) based solid-phase method with a C-terminal amide form, as previously described [56]. A cysteine (C) residue at the *N*-terminus was covalently bound to the MB group and two glycine (G) residues were used as a spacer between the cysteine and the active peptide sequence. More than a 10–100-fold excess amount of CGG peptide (against MB groups in chitosan) solution in 0.1% trifluoroacetic acid (TFA) was added to the MB–chitosan matrix-coated plates, and then an equal volume of 1% NaHCO_3_ solution was added to neutralize. After peptide coupling, the peptide–chitosan matrix was washed with phosphate-buffered saline two times and then with 0.1% BSA (bovine serum albumin) containing DMEM (Dulbecco’s modified essential medium) two times. Then, the peptide–chitosan matrices were blocked with 1% BSA in DMEM for 1 h, and biological activities were assessed using various cells.

## 3. Properties of Peptide–Chitosan Matrices

**Figure 2:**
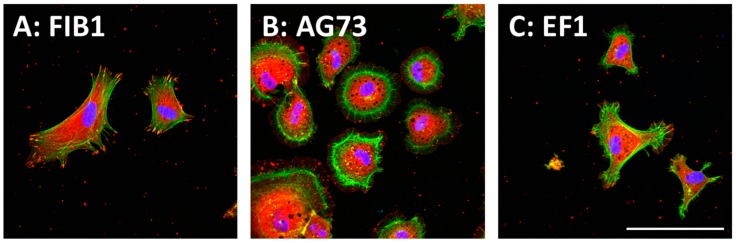
(**A**) CGG-FIB1; (**B**) CGG-AG73; and (**C**) CGG-EF1 peptides were covalently coupled to the MB–chitosan matrices (2 nmol/well) in 96-well plates. The amino acid sequences of three peptides are listed in Table 1. Human dermal fibroblasts (HDFs) were allowed to attach to the peptide–chitosan matrices for 2 h, and then actin (green), vinculin (red), and nucleus (blue) were observed by specific immunostaining. Scale bar is indicated 200 μm.

In the peptide–chitosan matrix, the chitosan matrix acts as a physical support, and the peptide interacts with the cells via specific cellular receptors. The ECM-derived peptide-conjugated chitosan matrices can promote receptor-type specific functions. We demonstrated that the cell attachment activity of peptides was enhanced by conjugation of the peptides to a chitosan matrix [36]. Cell attachment activity to the peptide–chitosan matrix depends on the amount of the immobilized peptides and is higher than that to peptide-coated plates [36,37,38]. These results suggest that the peptides maintain their active conformation on the chitosan matrix, and that the chitosan matrix is a suitable material for the cell attachment assay. The morphologies of the attached cells are depending on the peptides (Figure 2). Human dermal fibroblasts (HDFs) spread well on both FIB1 (YAVTGRGDSPAS, human FN, binds integrin α5β1, and αvβ3)–chitosan and EF1 (DYATLQLQEGRLHFMFDLG, mouse LM α1 chain, binds to integrin α2β1)–chitosan matrices with typical fibroblast-type actin stress fibers. In contrast, the AG73 (RKRLQVQSIRT, mouse LM α1 chain, binds to syndecans)–chitosan matrix promotes a round shape and induces the formation of actin filament spikes associated with membrane ruffles [57,58]. The morphological differences of cells are due to the interaction of the peptides with different cellular receptors (Table 1). Furthermore, the AG73–chitosan and FIB1–chitosan matrices promote neurite outgrowth but EF1–chitosan matrix does not have this activity [36,37,38].

RGD-containing peptides immobilized to chitosan have various cell attachment and proliferation activities. Rat osteosarcoma cells, bone stromal cells, and chondrocytes attached well to RGD–chitosan and promoted mineralization [33,53,59]. Further, Lv et al. reported that calcitonin gene-related peptide/chitosan containing cement promoted VEGF (vascular endothelial growth factor) mRNA expression and HUVEC (human umbilical vein endothelial cell) proliferation by controlling the release of the calcitonin gene-related peptide [60], and SIKVAV–chitosan hydrogels accelerated the re-epithelization of wounds and promoted angiogenesis in vivo [61].

Cell-transplantation is a useful medical application for tissue regeneration. We examined whether peptide–chitosan matrices are applicable as a scaffold material for cell-transplantation in vivo [62]. When human keratinocytes were seeded onto AG73–chitosan matrices, about 80% of the cells were attached to the matrices within 2 h. Then, the keratinocyte-AG73–chitosan matrix was inverted onto the artificial wound bed exposed on the back of a nude mouse. After three days, the human keratinocytes had migrated from the chitosan matrix and established a stratified epidermis-like structure on the mouse fascia. The transplanted cells expressed various keratinocyte markers, including cytekeratin-1, involucrin, and laminin γ2 chain, suggesting that the transplanted cells were undergoing cytodifferentiation resembling the epidermis. The peptide–chitosan matrix is useful as a scaffold material for keratinocyte delivery to the wound bed and the system has a potential to apply widely for cell-transplantation in vivo.

## 4. Cell Attachment Activities of Mixed Peptide–Chitosan Matrices Depending on Integrins and Syndecans

ECM proteins are multi-functional molecules and contain the many active sites. The regulation of receptor cross-talk by ECM proteins is not well defined because ECM proteins contain various cell attachment sites. Nam et al. reported that LM-111 peptides, A99 (AGTFALRGDNPQG) or YIGSR, were mixed and conjugated to a fibrin hydrogel, and then evaluated for the formation of lumen-containing parotid gland cell clusters. YIGSR-fibrin improved the morphology and lumen formation of parotid gland cells, and A99/YIGSR-fibrin promoted not only formation of functional three-dimensional cell clusters but also increased attachment and the number of cell clusters [31]. Yasa et al. reported that an IKVAV/RGD self-assembled peptide nanofiber supported adhesion, growth, and proliferation of the C2C12 myoblasts and significantly promoted the expression of skeletal muscle-specific marker genes [70].

The peptide–chitosan matrix can immobilize cell adhesive peptides with diverse mixing ratios and various mixed peptide–chitosan matrices have been demonstrated. Almost all peptide mixture work has been performed with the well-known cell adhesive peptides. One of the aims of our work is the identification of new biologically active peptides from ECM proteins based on a screen of the peptides conjugated to chitosan. For this purpose, a non-cell adhesive scaffold such as chitosan is suitable to identify the biological activities of peptides. The mixed peptide–chitosan matrices have been used to identify multiple biological functions and to mimic the biological activities of ECM proteins.

**Figure 3:**
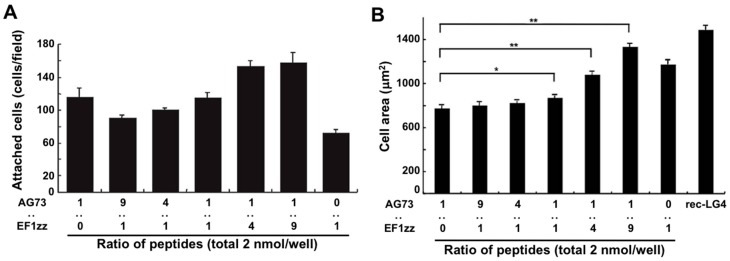
Cell attachment and spreading activity of mixed peptide–chitosan matrices. (**A**) CGG-AG73 and CGG-EF1zz peptides, both derived from the LM α1 chain LG45 globular module, were mixed in various ratios and were coupled to the MB–chitosan matrices (total peptide amount: 2 nmol/well) in 96-well plates. HDFs were allowed to attach to the mixed peptide–chitosan matrices for 2 h. Number of the attached HDFs in three randomly-selected fields was counted. Each value represents the mean ± S.D. of triplicate experiments. (**B**) HDFs were allowed to attach to either a recombinant LM α1 LG4 module protein (rec-LG4) (3 mg/well of protein solution was incubated overnight)-coated plate or peptide–chitosan matrices (2 nmol peptide/well) for 2 h. Cell images were captured, and the area of the attached cells were measured. Each value represents the mean ± S.D. of triplicate experiments. The AG73/EF1zz (1:9)–chitosan matrix promoted the strongest cell attachment and most extensive cell spreading comparable with that of rec-LG4. * *p* < 0.05, ** *p* < 0.005. (Adapted with permission from [68], copyright 2009 Elsevier).

The LM α1 chain contains five LM globular modules (LG1–LG5 modules) on its C-terminus, and the LM α1 LG4 and LG5 (LG45) module deleted mutant mouse does not survive beyond embryonic day E6.5 [71]. In the wild type mouse, the *N*-terminus of the LM α1 chain is condensed at Richert’s membrane in early embryonic stage and a proteolytic fragment of the LM α1 LG45 module is found at the ectoplacental cone that surrounds Richert’s membrane. This result indicates that the LM α1 LG45 module plays a critical role in mouse embryo development. We demonstrated that cell attachment to the recombinant LM α1 LG45 module protein (rec-LG45) is mediated by both syndecans and integrin α2β1 via the AG73 and EF1 sites, respectively [57]. At first, we focused on the LM α1 LG45 module and examined to mimic the full function of this domain using a mixed peptide–chitosan matrix system. We covalently conjugated the CGG-AG73 and CGG-EF1zz (a modified peptide of EF1, ATLQLQEGRLHFXFDLGKGR, X: Nle, binds to integrin α2β1) peptides to a chitosan matrix with various ratios (molar ratio = 1:0, 9:1, 4:1, 1:1, 1:4, 1:9, 0:1) and evaluated the biological activities [68]. The cell attachment activity and cell morphology depended on the ratios of AG73 and EF1zz on the chitosan matrix (Figure 3). Additionally, the AG73/EF1zz (molar ratio = 1:9)–chitosan matrix strongly promoted cell attachment, spreading, and neurite outgrowth similar to that on the rec-LG45. It was reported that cell attachment to the molecule is promoted by synergistic signaling between syndecans and integrins [68,72]. We found that the AG73/EF1zz (molar ratio = 1:9)–chitosan matrix contains an optimal peptide ratio for the synergistic effect of syndecans- and integrin α2β1-mediated cell attachment.

Further, to investigate the cross-talk between syndecans and other integrin subtypes, we prepared additional mixed peptide–chitosan matrices using two different integrin binding peptides, A99a (ALRGDN, a core sequence of A99, mouse LM α1 chain, binds to integrin αvβ3) and A2G10 (SYWYRIEASRTG, mouse LM α2 chain, binds to integrin α6β1) [66]. Either the combination of CGG-AG73/CGG-A99a or CGG-AG73/AGG-A2G10 were prepared with various mixture ratios, and conjugated on MB–chitosan matrix, respectively. The AG73/A99a (molar ratio = 1:10)–chitosan matrix showed three-fold enhanced cell attachment activity comparing with sum of the AG73–chitosan and A99a–chitosan matrix activities. The AG73/A2G10 (molar ratio = 1:25)–chitosan matrix showed two-fold enhanced cell attachment activity comparing with sum of the AG73–chitosan and A2G10–chitosan matrix activities. These results suggest that cross-talk between syndecans and integrins enhances cell attachment activity in the AG73/EF1zz (syndecans/integrin α2β1), AG73/A99a (syndecans/integrin αvβ3), and AG73/A2G10 (syndecans/integrin α6β1) mixtures.

Since cell morphology, migration, and stem cell differentiation are regulated by the rigidity of the scaffold, mechanosensing by the cells is important in cell-based tissue engineering [73]. Optimization of the scaffold stiffness has been noted as one of the bioengineering parameters for regulating cellular functions. We also determined whether density of the chitosan scaffold altered the cellular response [58]. When peptide A99a was conjugated to chitosan matrices of varying density (1.5–1500 ng/mm^2^), cell attachment was altered depending on the amount of chitosan. We found that 1.5–30 ng/mm^2^ of the A99a–chitosan matrix effectively promoted cell attachment, cell spreading with well-organized actin stress fibers, phosphorylation of focal adhesion kinase (FAK) Tyr397, and neurite outgrowth, but the higher density of A99a–chitosan matrix (150–1500 ng/mm^2^) reduced the activities. In contrast, AG73–chitosan matrix density (1.5–1500 ng/mm^2^) promoted similar biological activities at all of the concentrations tested. Further, when we examined the biological activity of AG73/A99a (molar ratio = 1:9)–chitosan matrix (1.5–1500 ng/mm^2^), higher density (150–1500 ng/mm^2^) of the AG73/A99a (molar ratio = 1:9)–chitosan matrix strongly promoted the biological activities. These results suggest that the mixed AG73/A99a–chitosan matrix effectively interacts with both integrin αvβ3 and syndecans on a stiffer chitosan matrix, and receptor interaction is sensitive to the scaffold density.

## 5. Cell Attachment Activities of Mixed Peptides–Chitosan Matrices Depending on Different Integrin Subtypes

**Figure 4:**
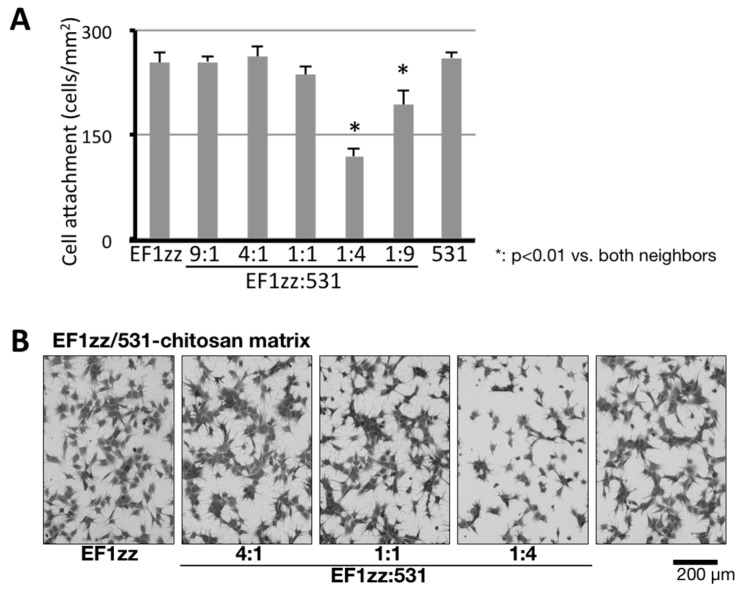
Specific suppression of HDF attachment on EF1zz/531–chitosan matrix. (**A**) CGG-EF1zz and CGG-531 peptides were mixed in various ratios and were coupled to the MB–chitosan matrices (10 nmol/well) in 96-well plates. HDFs were allowed to attach to the mixed peptide–chitosan matrices for 2 h. The attached HDFs in three randomly-selected fields were counted. Each value represents the mean ± S.D. of triplicate experiments. (**B**) EF1zz and 531 were mixed in various ratios (10:0, 4:1, 1:1, 1:4, and 0:10) and coupled to the MB–chitosan matrices, and HDFs (2 × 10^4^ cells/well) were allowed to attach to the mixed EF1zz/531–chitosan for 90 min and photographed. Scale bar indicates 200 μm. * indicates the *p* < 0.01 against EF1zz:531 = 1:4. (Adapted with permission from [63], copyright 2015 Elsevier).

We also investigated the cross-talk between different integrin-integrin subtypes. Integrins, transmembrane heterodimeric proteins comprising α and β subunits, are a major class of receptors for the ECM proteins and are involved in cell attachment [74,75]. Thus far, eighteen α and eight β subunits have been identified, and 24 different heterodimeric complexes have been determined as members of the integrin family. Integrins α1β1 and α2β1 mainly recognize COLs, integrins α5β1 and αvβ3 mainly recognize FNs, and integrins α3β1 and α6β1 mainly recognize LMs. When ECMs interact with integrins, biological functions are regulated through various mechanisms. Cell attachment to ECM proteins leads to integrin activation, which promotes intracellular signaling (outside-in) [74]. Outside-in integrin signaling controls the affinity of integrin binding to ECM proteins from a low-affinity state to a high-affinity state (inside-out) [74,76,77], with changes to the structure of integrins during cell migration and mechanotransduction. The outside-in or inside-out signaling is promoted not only by the same integrin subtype but also by different cellular receptors and different integrin subtypes [76,78]. We reported the suppression of cell attachment by an integrin-integrin cross-talk using mixed peptide–chitosan matrices (Figure 4) [63]. Two different integrin-binding peptides, EF1zz (binds to integrin α2β1) and 531 (GEFYFDLRLKGDKY, mouse COL type IV α1 chain, binds to integrin α3β1), were mixed at various molar ratios (9:1, 4:1, 1:1, 1:4, and 1:9) and conjugated on a chitosan matrix [63]. The EF1zz/531 (molar ratio = 1:4)–chitosan matrix exhibited significantly decreased cell attachment activity, whereas other EF1zz/531–chitosan matrices of different molar ratios did not show noticeable changes in activity, suggesting that integrin α2β1 and α3β1 have a suppressive cross-talk and the molar ratio is critical for the interaction. On the other hand, we recently focused on a different type of integrin-integrin cross-talk that promotes cell attachment activity (unpublished data). A mixed peptide–chitosan matrix conjugated integrin α5β1-, αvβ3-, and α6β1-binding peptides showed significantly increased cell attachment activity compared with sum of the peptide–chitosan matrices activities. These results suggest that the promotion of the synergistic effect between different integrin subtypes by mixing the different biologically active peptides on a chitosan matrix is a useful system to analyze the cross-talk of integrins.

## 6. Cell Attachment of Mixed Peptide–Chitosan Matrices Interacting with Multiple Receptors

Next, we focused on three or more cell surface receptor-binding peptides immobilized onto a chitosan matrix. The ECM contains a complex of multifunctional tissue-specific proteins in vivo. Thus, the interaction between cells and ECMs are simultaneously mediated by various cell surface receptors. For example, Lukjanenko et al. mixed various ECM proteins, such as FN, LM, type I COL, type IV COL, and vitronectin, in different proportions [79]. Interestingly, mouse skeletal muscle-derived myoblasts bind a FN and LM mixture, but human myoblast bind FN significantly. These results suggest that cell attachment mechanisms are different depending on the cells, the ECM matrix, and many cell surface receptors. To clarify these mechanisms, we developed LM-111–chitosan and FN–chitosan peptide matrices using mixtures of peptides attached to chitosan matrices as synthetic ECM biomaterials [64,65].

**Figure 5:**
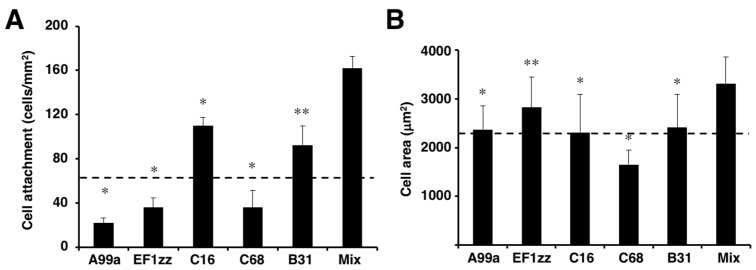
HDF attachment and spreading activity of peptide–chitosan matrices mimicking LM-111. CGG-A99a, CGG-EF1zz, CGG-C16, CGG-C68, and CGG-B31 (derived from LM-111, 10 nmol/well) both individually and as a five peptides mixture (Mix; 10 nmol/well; 2 nmol each) were coupled to the MB–chitosan matrices in 96-well plates. (**A**) HDFs were allowed to attach to the peptide and mixed peptide–chitosan matrices for 2 h. The attached cells in three randomly-selected fields were counted. Each value represents the mean ± S.D. of triplicate experiments. * *p* < 0.05; ** *p* < 0.005. (**B**) Cell areas on the various chitosan matrices were measured using Image J software (National Institutes of Health, Bethesda, MD, USA). The multipeptide–chitosan matrix promoted the most extensive cell spreading. (Adapted with permission from [65], copyright 2012 Elsevier).

Previously, we identified sixty biologically active peptides in the LM-111 sequence by a systematic peptide screening [65]. Twenty-nine distinct LM-111 derived peptide–chitosan matrices promoted various biological activities, including HDF attachment, spreading, and neurite outgrowth, and the activity depended on the peptide. We classified the twenty-nine biologically active peptides into six categories that indicated different biological activities based on cell attachment, cell surface receptors, and neurite outgrowth activity (Table 2). Five cell attachment peptides, including A99a (Group 1), EF1zz (Group 2), C16 (Group 3), C68 (Group 4, TSIKIRGTYSER, mouse LM γ1 chain), and B31 (Group 5, TNLRIKFVKLHT, mouse LM β1 chain), that showed the strongest activity in each category were mixed in equal amounts and conjugated onto a chitosan matrix to evaluate their synergistic activity (Figure 5). The mixed peptide (A99a/EF1zz/C16/C68/B31)–chitosan matrix significantly accelerated HDF attachment and cell spreading over that observed with any individual peptide (Figure 5). These results suggested that the five peptides cooperate and promote synergistic activity.

We mixed FIB1 (binds to integrins α5β1 and αvβ3) and ePRARI-C (QPPRARITGYII, human FN, binds to syndecan and integrin α4β1) and prepared a FIB1/ePRARI-C–chitosan matrix [64]. The FIB1/ePRARI-C–chitosan matrix was developed as a FN mimic biomaterial. The mixed ePRARI-C/FIB1 (molar ratio = 1:40) chitosan matrix promoted significantly better cell attachment and neurite outgrowth compared to that of ePRARI-C–chitosan and FIB1–chitosan matrices when added together. Cell attachment to the ePRARI-C/FIB1–chitosan matrix was mediated by integrins α4β1, α5β1, and αvβ3, similar to that of FN. These data suggest that the A99a/EF1zz/C16/C68/B31–chitosan and ePRARI-C/FIB1–chitosan matrices can be used as tools to analyze the multiple functions of LM-111 or FN and can serve as a LM-111- or FN-mimic biomaterial. The mixed peptide–chitosan matrix system using three or more receptor-specific binding peptides has various advantages and is a powerful tool for evaluating the mechanism of the multi-receptor interactions, including cell-ECM interactions.

## 7. Neurite Outgrowth Activities of Mixed Peptide–Chitosan Matrices

Chitosan structured as a hollow tube has been use as a guide for the neurite growth [80]. Chitosan tubes have been used to reconstitute peripheral nerves and regenerate injured rodent transected sciatic nerve with results comparable to that of autologous nerve graft repair [81,82]. Implantation of a chitosan nanofiber tube could partially restore the function of a damaged phrenic nerve in beagle dogs as seen by improvement in diaphragm movement, slow phrenic nerve conduction, and connection of the damaged nerve with the newly regenerating nerve fibers surrounded by granulation tissue within the chitosan nanofiber tube [83]. Furthermore, ECM proteins, including COLs, LMs, and FN, are key molecules that promote neurite outgrowth in vivo [23,84]. Several ECM-derived peptides have been found to promote neurite outgrowth [65,85]. In a neurite outgrowth assay in vitro, long-term cell culture (more than 24 h) is required to evaluate neurite outgrowth activity. A peptide–chitosan matrix is stable compared when compared to peptide alone and is applicable for long-term cell culture.

**Figure 6:**
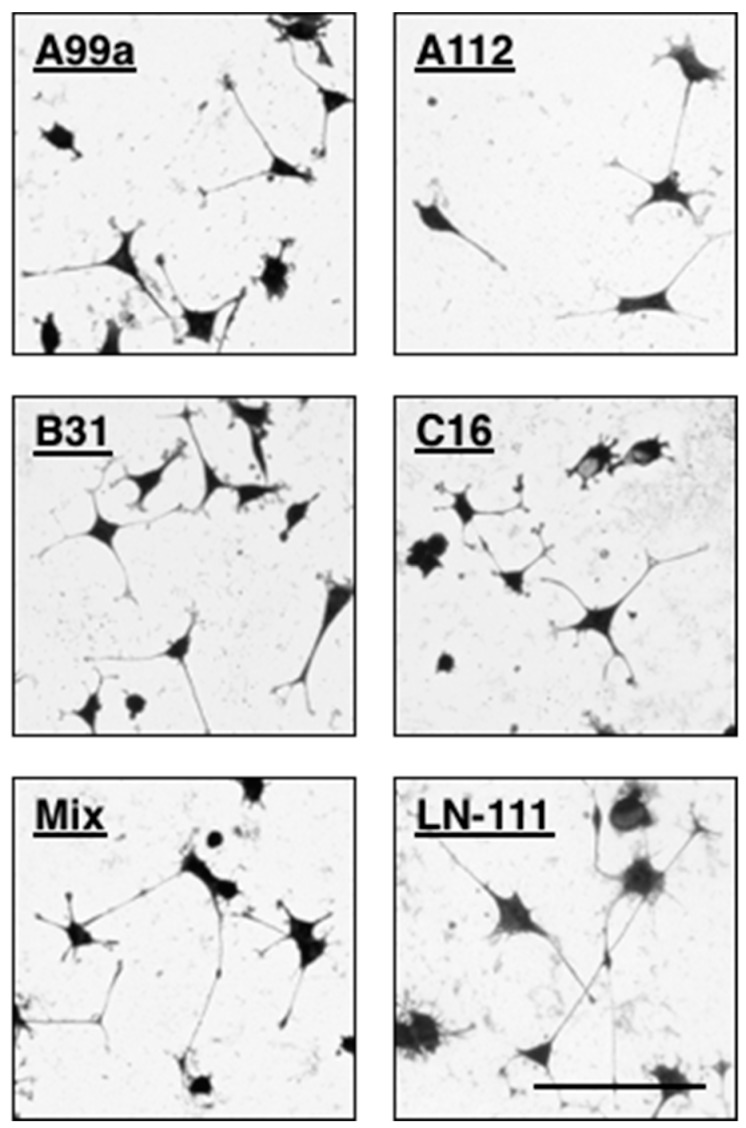
Neurite outgrowth activity of multipeptide–chitosan matrices mimicking LM-111. CGG-A99a, CGG-A112, CGG-C16, and CGG-B31 (10 nmol/well), and four peptides mixture (each peptide 2.5 nmol/well; total peptide 10 nmol/well) were coupled to the MB–chitosan matrices in 96-well plates. The PC12 cells were allowed to incubate on the peptide–chitosan matrices for 24 h and then stained with crystal violet. Scale Bar is indicated 100 µm. (Adapted with permission from [65], copyright 2012 Elsevier).

The peptide–chitosan matrix system has an advantageous for neurite outgrowth assay. As shown in above (Section 4), the AG73/EF1zz (molar ratio = 1:9)–chitosan matrix strongly promoted neurite outgrowth. The neurite outgrowth activities of AG73/EF1zz (molar ratio = 1:9)–chitosan matrix was stronger than that of the AG73–chitosan and EF1zz–chitosan matrices and similar to that on the rec-LG45. The mixed peptide–chitosan system is also useful to design a biomaterial for neuronal cell differentiation. Interestingly, some peptides from laminin have PC12 cell neurite outgrowth activity but not fibroblast adhesion activity (Table 2; Group 6) [65]. PC12 cell neurite outgrowth of mixed peptide–chitosan matrices using laminin-111 derived peptides has been described. We mixed four peptides that promote neurite outgrowth from each group (Table 2), including A99a (Group 1), C16 (Group 3), B31 (Group 5), and A112 (Group 6, VLIKGGRARKHV, mouse LM α1 chain), and prepared a mixed peptide (A99a/C16/B31/A112)–chitosan matrix. The mixed (A99a/C16/B31/A112)–chitosan matrix showed stronger neurite outgrowth activity compared with that observed on each individual peptide–chitosan matrix (Figure 6). The neurites on each single peptide–chitosan matrix were relatively short, wide, and curved. In contrast, neurites on the mixed peptide–chitosan matrix and LM-111 were long, thin, and linear. Synergistic cooperation among the different cellular receptors was observed on the multi-peptide matrices, and the activity was similar to that of the intact ECM protein.

## 8. Summary and Outlook

**Figure 7:**
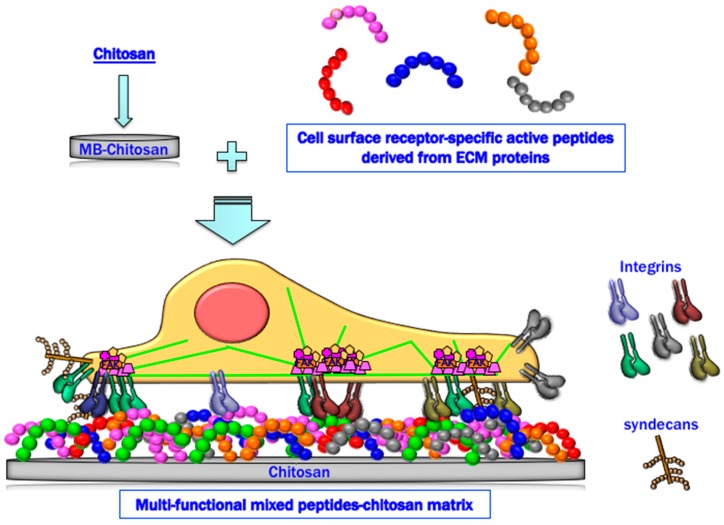
Schematic representation of a mixed peptide–chitosan matrix system. The mixed peptide–chitosan matrix using different receptor-binding peptides may elicit simultaneous cellular interactions and reproduce the biological activity of the intact ECM proteins.

In summary, peptide–chitosan matrices specifically interact with cellular receptors and promote various biological activities. The mixed peptide–chitosan matrix system using receptor-specific peptides can reproduce the multi-receptor interactions and synergistically enhance the biological activities with their receptor cross-talk (Figure 7). Based on these results, we conclude that the mixed peptide–chitosan matrix using different receptor-binding peptides can elicit simultaneous cellular interactions and could reproduce the molecular functions of the intact ECM molecule. The mixed peptide–chitosan matrix system is a useful strategy to develop biomaterials as a synthetic ECM.

## Figures and Tables

**Table 1:** List of cell adhesive peptides and their characters.

Peptides	Sequence ^a^	Original Proteins	Receptors	References
531	CGG-GEFYFDLRLKGDKY	COL4-α1	Integrin α3β1	[63]
FIB1	CGG-YAVTGRGDSPAS	FN	Integrin αvβ3 Integrin α5β1	[64]
ePRARI-C	CGG-QPPRARITGYII	FN	Integrin α4β1 syndecans	[64]
A99	CGG-AGTFALRGDNPQG	LM-α1	Integrin αvβ3	[58,65,66]
A112	CGG-VLIKGGRARKHV	LM-α1	N.D. ^b^	[65]
AG73	CGG-RKRLQVQSIRT	LM-α1	syndecans	[57,58,65,66,67,68]
EF1	CGG-DYATLQLQEGRLHFXFDLG	LM-α1	Integrin α2β1	[57]
EF1zz	CGG-ATLQLQEGRLHFXFDLGKGR X:Nle, a modified EF1	LM-α1	Integrin α2β1	[63,65,67,68]
A2G10	CGG-SYWYRIEASRTG	LM-α2	Integrin α6β1	[66,69]
B31	CGG-TNLRIKFVKLHT	LM-β2	syndecans	[65]
C16	CGG-KAFDITYVRLKF	LM-γ1	Integrin β1 syndecans	[65,66]
C68	CGG-TSIKIRGTYSER	LM-γ1	N.D. ^b^	[65]

^a^ Cys-Gly-Gly (CGG) sequence was added at the *N*-terminus of the peptides. The C was for conjugation to MB–chitosan, and the GG was a spacer between the peptide and chitosan. All peptides contain C-terminal amide; ^b^ N.D.: not determined.

**Table 2:** Twenty-nine peptide–chitosan matrices could be divided into six categories depending on their biological activities.

Group	Biological Activities on HDFs ^a^					PC12 Cell N.O. ^c^	Peptides
	Attachment	Spreading ^b^	Inhibitory Effect		Receptors		
			EDTA	Heparin			
1	+	S	+	−	Integrin	+	A99a
2	+	S	+	−	Integrin	−	EF1zz
3	+	s	+	+	Integrin/ syndecan	+	A13, AG32, AG103, C16, C57, C64
4	+	s	+	+	Integrin/ syndecan	−	A3, A55, A65, A119, A167, A174, AG10, AG28, AG56, B30, B133, B160, C59, C68
5	+	w	−	+	Syndecan	+	A206, AG73, B20, B31
6	−	−	−	−	−	+	A25, A112, A194

Twenty-nine peptide–chitosan matrices using laminin-111 active peptides were divided into six groups depending on their biological activities. ^a^ Biological activities of HDF attachment and neurite outgrowth indicate as + (active) or − (inactive). Inhibitory effect of either EDTA or heparin on HDF attachment indicate as + (reduce attachment) or − (no change). ^b^ HDF spreading: S, extended spreading; s, moderate spreading; w, wide and round spreading. ^c^ Neurite outgrowth activities of PC12 cells on peptide–chitosan matrices. (Adapted with permission from [65], copyright 2012 Elsevier).
